# Long-Term Clinical Course of Normal-Tension Glaucoma: 20 Years of Experience

**DOI:** 10.1155/2017/2651645

**Published:** 2017-04-05

**Authors:** Sang Wook Jin, Seung Yoon Noh

**Affiliations:** Department of Ophthalmology, Dong-A University College of Medicine, Busan, Republic of Korea

## Abstract

*Purpose*. The purpose of this study was to investigate the long-term clinical course of NTG patients who initiated intraocular pressure- (IOP-) lowering therapy. *Methods*. The present study included 72 normal-tension glaucoma (NTG) patients. The mean deviation (MD) was measured with visual fields. Nocturnal hypotension with weighted standard deviation (wSD) was determined by 24-hour ambulatory blood pressure monitoring. To identify risk factors for NTG progression, linear logistic regression analysis was employed. *Results*. The mean follow-up period was 21.2 ± 1.1 years. The mean MD progression rate was −0.28 ± 0.24 dB/year. The mean ocular perfusion pressure (OPP) was 52.1 ± 5.9 mmHg. The mean wSD was 14.5 ± 2.2. In the univariate model, disc hemorrhage (RR 7.12; *P* = 0.004), IOP reduction rate (RR 2.12; *P* = 0.045), and OPP (RR 1.94; *P* = 0.027) were associated with glaucomatous visual field progression. However, in the multivariate model, the IOP reduction rate (RR 2.45; *P* = 0.048) and OPP (RR 2.02; *P* = 0.004) were detected to be significant factors associated with progression. *Conclusions*. The mean rate of visual field progression was −0.28 dB/year in NTG patients treated with medical therapy. The IOP reduction rate and OPP were associated with glaucomatous visual field progression.

## 1. Introduction

Glaucoma describes a group of optic neuropathies that result in the progressive loss of ganglion cells. It manifests as characteristic optic disc cupping, nerve fiber layer loss, and visual field defects [1].

Normal-tension glaucoma (NTG) is more prevalent than high-tension glaucoma in Asia. In addition, a recent population-based study reported that the most common type of glaucoma in Koreans was NTG [2, 3].

NTG is a widely known multifactorial disease in pathogenesis and disease progression. Although the pathogenesis and disease progression in NTG has not been fully elucidated, several factors affecting disease progression have been found [4–6].

Many risk factors for the development and progression of NTG have been identified, of which intraocular pressure (IOP) is considered the most important modifiable factor [7, 8]. However, IOP alone can explain only a small proportion of the pathogenesis of glaucoma [9].

IOP independent factors, such as vascular factors, can lead to hypoperfusion of the optic disc head. And consequently glaucomatous optic disc change has occurred [10–12]. Among several vascular factors, low BP, BP variability, nocturnal hypotension, and low or fluctuating ocular perfusion pressures (OPP) represented major risk factors for the prevalence, incidence, and progression of glaucoma in large epidemiological surveys [13–16].

Clinical data about the disease, such as the rate of glaucomatous visual field defect progression and risk factors for disease progression, might be helpful to clinicians to estimate prognosis and devise treatment regimens.

The representative, population-based, cross-sectional studies of early open-angle glaucoma were those of the Collaborative Normal-Tension Glaucoma Study (CNTGS) [7] and Early Manifest Glaucoma Trial (EMGT) [8]. These data suggested the rates of glaucomatous visual field progression or the natural course of NTG. However, the average follow-up period of these studies was approximately 12 years, which was insufficient to represent the long-term clinical course of NTG.

Because of the lack of long-term follow-up data, we investigated the long-term clinical courses of NTG patients who initiated IOP-lowering therapy. In addition, this study evaluated the risk factors, including vascular factors for glaucomatous visual field defect progression.

## 2. Methods

### 2.1. Subjects

This study was approved by the Institutional Review Board of Dong-A University. Informed consent was obtained from each participant, and all of the study conduct adhered to the tenets of the Declaration of Helsinki.

The medical records of patients who had been diagnosed and followed up for NTG from 1994 to 2015 at Dong-A University Hospital were retrospectively examined.

At the initial glaucoma evaluation, each patient underwent a comprehensive ophthalmologic examination, including a review of the patient's medical history, measurement of best-corrected visual acuity (BCVA), IOP, gonioscopy, central corneal thickness (CCT) measurement, dilated funduscopic examination, retinal nerve fiber layer (RNFL) photography, and standard automated perimetry using a 24-2 Swedish Interactive Threshold Algorithm (SITA; Carl Zeiss Meditec, Dublin, CA, USA).

NTG was diagnosed as glaucomatous optic neuropathy using funduscopic examination, characteristic visual field defects, open anterior chamber angles on gonioscopy, and pretreatment IOP never exceeding 21 mmHg, as measured by the Goldmann applanation tonometer (GAT). IOP was tested by the same examiner during the day, and the average of the three measurements was used in the analyses.

The visual field test was performed on patients whose follow-up was at least 5 years, which equated to more than 10 follow-ups with a minimum 6-month interval. Eyes with glaucomatous visual field defects were defined as those that met two of the following criteria as confirmed by more than two reliable consecutive tests, in addition to compatibility with optic nerve appearance: (1) a cluster of three points with a probability of less than 5% on a pattern deviation map in at least one hemifield and including at least one point with a probability of less than 1% or a cluster of two points with a probability of less than 1%; (2) a glaucoma hemifield test (GHT) result beyond 99% of the age-specific normal limit; and (3) a pattern standard deviation (PSD) beyond 95% of the normal limit. Reliable visual field assessment was defined as a visual field test with a false-positive error < 15%, a false-negative error < 15%, and a fixation loss < 20%. The first perimetric result was excluded from the analysis to obviate learning effects.

Mean deviation (MD) value was used to determine visual field progression. Glaucomatous visual field progression was defined as one of the following findings: (1) significant deterioration from the baseline pattern deviation at three or more test points that were evaluated on three consecutive examinations or as a significantly negative slope (*P* < 0.05) in linear regression analysis using the mean deviation (MD) data (criteria A) [8] and (2) an annual decrease in MD slope of less than 0.5 dB/year (criteria B) [17]. The rate of progression was determined according to the slope of the linear regression analysis of MD values over time.

The 24 hr ABPM was performed using an electronic sphygmomanometer (TONOPORT V., GM Medical System, Germany) on the patient's nondominant arm. Daytime BP (7 AM to 10 PM) was measured at 30-minute intervals, and nighttime BP was measured (10 PM to 7 AM) at one-hour intervals. The patient was allowed to lead a normal active life as much as possible during the monitoring of ambulatory blood pressure.

MAP is calculated as [18]

(1) $$\begin{array}{c} MAP=diastolic\;BP+\frac{1}{3}\;\left(systolic\;BP-diastolic\;BP\right). \end{array}$$

OPP is calculated as [19]

(2) $$\begin{array}{c} OPP=\frac{2}{3}\;MAP-IOP. \end{array}$$

Nocturnal hypotension represents how much the average value of nighttime BP decreases compared to the average of daytime BP, and it can be calculated using the following equation:

(3) $$\begin{array}{c} Nocturnal\;hypotension=\frac{\left(avarage\;of\;daytime\;MAP-avarage\;of\;nighttime\;MAP\right)}{avarage\;of\;daytime\;MAP}\times 100. \end{array}$$

Based on the above equation, patients with <10% nocturnal hypotension were defined as “nondippers,” those with ≥10% but <20% nocturnal hypotension were defined as “dippers,” and those with ≥20% nocturnal hypotension were defined as “overdippers” [15, 20].

BP variability was represented by weighted standard deviation (wSD), which can be calculated using the following equation [21]:

(4) $$\begin{array}{c} \text{weighted standard deviation }\left(wSD\right)=\frac{\left(daytime\;SD\times daytime\;valid\;measurement\right)+\left(nighttime\;SD\times nighttime\;valid\;measurement\right)}{all\;time\;valid\;measurement}. \end{array}$$

Patients were included in the study if they fulfilled the following criteria: (1) newly diagnosed early NTG; (2) a follow-up period of more than 20 years; (3) a visual field test performed on patients whose follow-up lasted at least 5 years, which equated to more than 10 follow-ups with a minimum 6-month interval; and (4) 24 hr ambulatory blood pressure monitoring (24 hr ABPM) performed on patients whose follow-up lasted at least 5 years, which equated to more than 5 follow-ups with a minimum 12-month interval.

The exclusion criteria were as follows: (1) eyes with other visually significant ocular pathology (e.g., visually significant cataracts, diabetic retinopathy, vascular occlusions, and macular degeneration); (2) patients on medications (e.g., steroids, hydroxychloroquine) that could affect visual sensitivity and IOP; (3) a history of ocular surgery, including cataract operations; (4) any significant medical problems with ocular manifestations, such as diabetes, hypertension, and other systemic diseases that might result in a visual field defect; (5) improper recording of the timing of IOP measurements during the follow-up periods; and (6) failure to attend outpatient visits regularly.

We also excluded patients using beta-blockers or dorzolamide antiglaucoma eye drops because of the systemic effects of eye drops on blood pressure or ocular blood flow.

One eye from each subject was used for the analysis, and when both eyes had the same glaucoma diagnosis and visual field progression, the right eyes were used for the analysis.

### 2.2. Statistical Analysis

Statistical analyses were performed using the SPSS software program (version 20.0; SPSS Inc., Chicago, IL, USA). Categorical variables were investigated by cross-tables and the chi-square test. Student's paired *t*-test or the Mann-Whitney *U* test was used for the analysis of continuous variables. Univariate and multivariate logistic regression analyses were used to identify the risk factors for glaucomatous visual field progression. One-way analysis of variance (ANOVA) was performed to compare the three groups, and Bonferroni's test was performed for post hoc comparisons. *P* values less than 0.05 indicated statistical significance.

## 3. Results

A total of 158 NTG patients were followed over 20 years. Of these 115 patients, 72 eyes of 72 NTG patients (62.6%) were enrolled in the study and 43 NTG patients (37.4%) were excluded. The reasons for exclusion were as follows: (1) lack of total number of 24 hr ABPM exam (16 patients, 16 eyes); (2) inadequate result of 24 hr ABPM exam (11 patients, 11 eyes); (3) received cataract surgery during the follow-up period (9 patients, 9 eyes); and (4) usage of beta-blockers or dorzolamide eye drops during the follow-up period (7 patients, 7 eyes). All of the enrolled patients were Koreans. The mean age at initial examination was 58.4 ± 12.4 years old, and the mean follow-up period was 21.2 ± 1.1 years. The central corneal thickness averaged 535.4 ± 13.2 *μ*m. The mean baseline IOP was 16.6 ± 3.1 mmHg, the mean IOP after IOP-lowering therapy was 11.9 ± 2.2 mmHg, and the average reduction rate of IOP was 28.3%. Baseline MD was −3.59 ± 2.21 dB. The mean MD progression rate was −0.28 ± 0.24 dB/year. The mean OPP was 52.1 ± 5.9 mmHg. The mean rate of systolic nocturnal hypotension was 7.6 ± 4.5%, and that of diastolic nocturnal hypotension was 8.5 ± 5.9%. The mean wSD was 14.5 ± 2.2 (Table 1).

The mean MD progression rate of all of the patients was −0.28 ± 0.24 dB/year. Rates of visual field progression between 0 dB/year and 0.5 dB/year were observed in 9.7% (7/72), rates from 0 dB/year to −0.5 dB/year were observed in 73.6% (53/72), rates from −0.5 dB/year to −1.0 dB/year were observed in 11.1% (8/72), and rates greater than −1.0 dB/year were observed in 5.6% (4/72).

Among the 72 NTG patients, 28 patients (38.9%) showed glaucomatous visual field progression. Of these 28 patients, 11 patients (11 eyes, 15.3%) showed significant glaucoma visual field progression according to criteria A, and 10 patients (10 eyes, 13.9%) showed significant glaucoma visual field progression. Seven patients (7 eyes, 9.7%) showed glaucomatous visual field progression by both criteria A and B.

Results of the logistic regression analysis identified factors associated with glaucomatous visual field progression results, which are presented in Table 2. In the univariate model, disc hemorrhage (RR 7.12; *P* = 0.004), IOP reduction rate (RR 2.12; *P* = 0.045), and OPP (RR1.94; *P* = 0.027) were associated with glaucomatous visual field defect progression. In the multivariate model, IOP reduction rate (RR 2.45; *P* = 0.048) and OPP (RR 2.02; *P* = 0.004) were detected to be significant factors associated with progression (Table 2).

Comparison results of the clinical characteristics among the three groups (nondippers, dippers, and overdippers) are shown in Table 3. In the nondippers group, the mean progression rate was −0.20 ± 0.21 dB/year, OPP was 52.3 ± 6.1, and wSD was 14.0 ± 2.0. In the dippers group, the mean progression rate was −0.24 ± 0.20 dB/year, OPP was 51.7 ± 4.7, and wSD was 13.9 ± 2.1. In the overdippers group, the mean progression rate was −0.28 ± 0.30 dB/year, OPP was 46.2 ± 5.4, and wSD was 15.3 ± 1.3 (Table 3).

## 4. Discussion

Previous population-based research has suggested rates of visual field progression. The CNTGS reported that annual decrease in MD was −2 dB in NTG [7]. The EMGT reported that the mean rate of visual field defect progression in untreated NTG patients was 0.36 dB/year [8]. Broman et al. [22] reported that the mean worsening of visual fields in Chinese POAG patients was −1.56 dB/year. Komori et al. [17] also reported that the mean visual field progression rate in Japanese NTG patients was −0.30 dB/year. In our study, the mean rate of visual field progression was −0.28 dB/year in treated NTG patients. This result was similar to previous studies. However, despite achieving an almost 30% reduction rate in IOP by medical therapy, 26.4% of treated NTG patients experienced progressed glaucomatous visual field defects. Therefore, when making decisions for glaucoma management, clinicians should consider mechanisms other than IOP-dependent factors that might contribute to glaucomatous visual field progression.

The risk factors for glaucomatous visual field progression or poor prognosis have been reported in previous studies, including IOP, disc hemorrhage, myopia, age, low blood pressure, nocturnal hypotension, migraine, Raynaud's phenomenon, and sleep apnea [7, 8, 23–27].

In the present study, IOP reduction rate and OPP were found to be risk factors for glaucomatous visual field progression. However, disc hemorrhage, which is a well-known risk factor for glaucoma progression in previous studies, was not detected to be a risk factor in multivariate analysis.

The importance of IOP reduction rate has been emphasized in many previous studies [7, 8, 28]. In the CNTGS [7], visual field defect progression was more common in the untreated group than in the treated group (30% IOP reduction from baseline). In the EMGT [8], risk decreased by approximately 10% with each mmHg of IOP reduction from baseline. The result of our present study that IOP reduction rate was associated with glaucomatous visual field defect progression was consistent with previous studies.

OPP is a well-known and important factor of disease progression in NTG [8, 29]. In EMGT [8], lower systolic OPP was confirmed to be a significant predictive factor for glaucoma progression. Sung et al. [29] reported that higher levels of 24 hr mean OPP fluctuation were associated with greater glaucoma visual field defect progression. In the present study, lower levels of OPP were a significant risk factor for glaucoma visual field progression other than systemic blood pressure. As a result, low OPP could lead to ischemic changes in the optic nerve head and glaucomatous visual field defect progression.

Among the IOP-independent risk factors for NTG progression, vascular factors play important roles in disease progression [10, 14–16]. We divided the patients into 3 groups as nondippers, dippers, and overdippers to evaluate the effects of vascular factors on disease progression. Compared to the nondippers and dippers, overdippers showed a worse MD slope, lower OPP, and higher blood pressure variability. This result suggested that, even in NTG patients who achieved target IOP, nocturnal hypotension over the physiological dip and large variation in blood pressure led to progression of glaucomatous visual field defects. Therefore, these patients might be considered for correction of severe nocturnal hypotension and large variability in blood pressure.

This study had the following limitations. First, this study was a retrospective study and had a high rate of exclusion; there might have been selection bias. Second, the sample size was small. Therefore, we believe that additional studies are needed. Third, we have evaluated the glaucoma progression using visual field criteria only. Further studies, evaluating glaucoma progression using both optic nerve head and retinal nerve fiber layer changes, are needed. Fourth, OPP was calculated with an indirect method by brachial BP; therefore, our OPP data were not actual OPP. Finally, our results did not reflect other risk factors of NTG progression, such as diurnal IOP fluctuation. However, we believe that our study provided clinicians useful long-term clinical data about NTG and the risk factors for NTG progression.

In conclusion, the mean rate of visual field progression was −0.28 dB/year in NTG patients treated with medical therapy. However, despite achieving an almost 30% reduction rate in IOP by medical therapy, disease progression occurred in some cases. Therefore, clinicians should consider mechanisms other than IOP-dependent factors that might contribute to glaucomatous visual field progression. Concerning the analysis of risk factors for NTG progression, adequate IOP reduction and correction of low OPP might help to slow or stop NTG progression.

## Figures and Tables

**Table 1:** Baseline demographics and characteristics of study participants.

Characteristic	
M : F (*N*)	32 (44.4%) : 40 (55.6%)
Age (years)	58.4 ± 12.4
Mean follow-up period (years)	21.2 ± 1.1
CCT (mmHg)	535.4 ± 13.2
Mean baseline IOP (mmHg)	16.6 ± 3.1
Mean IOP (mmHg)	11.9 ± 2.2
Mean reduction rate of IOP (%)	28.3%
Baseline MD of visual field (dB)	−3.59 ± 2.21
MD slope (dB/year)	−0.28 ± 0.24
OPP	52.1 ± 5.9
Nocturnal hypotension (systolic/diastolic) (%)	7.6 ± 4.5/8.5 ± 5.9
wSD	14.5 ± 2.2

Expressed as the mean ± SD; CCT: central corneal thickness; IOP: intraocular pressure; MD: mean deviation; OPP: ocular perfusion pressure; wSD: weighted standard deviation.

**Table 2:** Logistic regression analysis of the association between the clinical parameter and glaucomatous visual field progression.

Variables	Univariate model RR (95% CI)	*P* value	Multivariate model RR (95% CI)	*P* value
Female sex	1.97 (0.52–4.39)	0.859	2.04 (0.50–5.32)	0.442
Age	1.27 (0.78–2.12)	0.682	1.12 (0.75–3.02)	0.248
CCT	0.34 (0.12–1.92)	0.897	0.42 (0.22–2.93)	0.617
Baseline IOP	0.87 (0.71–1.82)	0.537	0.57 (0.41–1.52)	0.313
Mean IOP	0.77 (0.67–1.54)	0.265	0.92 (0.57–1.44)	0.331
IOP reduction rate	1.12 (0.95–2.92)	**0.045**	1.45 (0.83–2.43)	**0.048**
Disc hemorrhage	7.12 (4.57–13.29)	**0.004**	8.11 (3.53–16.33)	0.051
Baseline MD	1.02 (0.88–2.36)	0.518	1.56 (0.83–2.39)	0.501
Sys. nocturnal hypotension	0.69 (0.52–1.54)	0.248	0.73 (0.42–1.64)	0.243
Dia. nocturnal hypotension	1.25 (1.01–3.92)	0.254	1.15 (0.95–4.12)	0.319
OPP	1.94 (0.97–3.12)	**0.027**	2.02 (0.84–3.92)	**0.004**
wSD	1.01 (0.71–1.78)	0.362	1.32 (0.61–2.15)	0.321

Expressed as the mean ± SD; bolded *P* values indicate statistical significance; RR: relative risk; CI: confidence interval; CCT: central corneal thickness; IOP: intraocular pressure; MD: mean deviation; Sys.: systolic; Dia.: diastolic; OPP: ocular perfusion pressure; wSD: weighted standard deviation.

**Table 3:** Comparisons among 3 groups (nondippers, dippers, and overdippers).

	Nondippers	Dippers	Overdippers	*P* value
MD slope (dB/y)	−0.20 ± 0.21	−0.24 ± 0.20^§^	−0.28 ± 0.30^†‡^	**0.000** ^∗^
MAP (mmHg)	95.1 ± 4.4	94.6 ± 3.3	90.7 ± 5.9^†‡^	**0** **.000** ^∗^
OPP (mmHg)	52.3 ± 6.1	51.7 ± 4.7	46.2 ± 5.4^†‡^	**0.000** ^∗^
wSD	14.0 ± 2.0	13.9 ± 2.1	15.3 ± 1.3^†‡^	**0.014** ^∗^

Expressed as the mean ± SD; ^∗^comparison among 3 groups by one-way analysis of variance; statistical significance: *P* < 0.05; ^†^significantly different compared with nondippers by post hoc multiple comparison (Bonferroni's test); ^‡^significantly different compared with dippers by post hoc multiple comparison (Bonferroni's test); ^§^significantly different compared with nondippers by post hoc multiple comparison (Bonferroni's test); MD: mean deviation; MAP: mean arterial pressure; OPP: ocular perfusion pressure; wSD: weighted standard deviation.
